# Pain Processing in a Social Context and the Link with Psychopathic Personality Traits—An Event-Related Potential Study

**DOI:** 10.3389/fnbeh.2017.00180

**Published:** 2017-09-25

**Authors:** Casper H. van Heck, Josi M. A. Driessen, Maria Amato, Marnou N. van den Berg, Pritha Bhandari, Laura Bilbao-Broch, Jordi Farres-Casals, Manon Hendriks, Adrian C. Jodzio, Laura Luque-Ballesteros, Christina Schöchl, Laura R. Velasco-Angeles, Roel H. A. Weijer, Clementina M. van Rijn, Marijtje L. A. Jongsma

**Affiliations:** ^1^Donders Institute for Brain, Cognition, and Behavior, Donders Center for Cognition, Radboud University Nijmegen Nijmegen, Netherlands; ^2^Radboud University Medical Centre, Cognitive Neuroscience Nijmegen, Netherlands; ^3^Behavioural Science Institute, Radboud University Nijmegen Nijmegen, Netherlands

**Keywords:** electrophysiology, ERP, pain, psychopathy, empathy

## Abstract

Empathy describes the ability to understand another person’s feelings. Psychopathy is a disorder that is characterized by a lack of empathy. Therefore, empathy and psychopathy are interesting traits to investigate with respect to experiencing and observing pain. The present study aimed to investigate pain empathy and pain sensitivity by measuring event-related potentials (ERPs) extracted from the ongoing EEG in an interactive setup. Each participant fulfilled subsequently the role of “villain” and “victim”. In addition, mode of control was modulated resulting in four different conditions; passive villain, active villain, active victim and passive victim. Response-, visual- and pain ERPs were compared between the four conditions. Furthermore, the role of psychopathic traits in these outcomes was investigated. Our findings suggested that people experience more conflict when hurting someone else than hurting themselves. Furthermore, our results indicated that self-controlled pain was experienced as more painful than uncontrolled pain. People that scored high on psychopathic traits seemed to process and experience pain differently. According to the results of the current study, social context, attention and personality traits seem to modulate pain processing and the empathic response to pain in self and others. The within-subject experimental design described here provides an excellent approach to further unravel the influence of social context and personality traits on social cognition.

## Introduction

### Pain and Empathy

From an evolutionary point of view, pain signals actual or potential injury or damage to bodily parts and is thereby a protective mechanism. Perceived pain severity can be greatly influenced by various factors, such as attention and expectancy (Melzack and Wall, 1967). Moreover, it has been determined that pain is perceived as less intense when it is self-controlled (Pellino and Ward, 1998; Salomons et al., 2004).

Humans are naturally social individuals and experience discomfort while observing another person in pain. This phenomenon, termed as “pain empathy”, is a complex construct that describes the ability to understand another person’s situation or feelings (Davis, 1980; Lietz et al., 2011) and is believed to be one of the requirements for successful participation in current society (Schneider and Ingram, 2005). Neuroimaging studies focussing on empathy received considerable effort in the past decade (Singer and Lamm, 2009; Decety, 2010). For instance, previous studies showed that ongoing information processing is affected differently when being exposed to pictures that show another person’s pain than being exposed to neutral pictures (Avenanti et al., 2005; Bufalari et al., 2007). Evidence from neuroimaging research suggests that experienced pain and observed pain in others elicit similar activation patterns in brain areas involved in the processing of both affective (e.g., the anterior insula and the medial/anterior cingulate cortex (ACC; Decety, 2010)) and sensory (e.g., the primary somatosensory cortex and parietal operculum (Bufalari et al., 2007)) information. These findings support the theory that describes a shared neural network for one’s own and others’ emotional and sensory experience.

Current models of pain empathy suggest that empathy-related processes are derived from both bottom-up features and top-down factors (Decety and Moriguchi, 2007). Zooming in on these top-down factors, social context seems to be an important modulator of pain perception in self and others (Singer et al., 2006; Decety et al., 2009). Several aspects of social context, such as relationships between individuals (Singer et al., 2006) and attitude towards others (Decety et al., 2009) have been studied previously. Studying the lack of empathy with respect to pain might be even more salient.

### Psychopathy; a Pain- and Empathy-Related Disorder

Psychopathy is a disorder that is linked to deviant pain processing and experience. Although the majority of studies have been focused on psychopathy in criminal offenders (Thompson et al., 2014), psychopathic personality traits are demonstrated to be normally distributed in the general population (Levenson et al., 1995; Hare and Neumann, 2008; Gao and Raine, 2010). Recent neuroimaging studies have suggested that an attenuated function in the amygdala and anterior insula underlies reduced empathy in individuals with high levels of psychopathic traits (Seara-Cardoso et al., 2015). Moreover, research revealed that people high in psychopathic traits show atypical neural activity in response to imagining others’ pain (Decety et al., 2013; Seara-Cardoso et al., 2015). Besides characteristics of lack of empathy, psychopaths tend to experience pain differently compared to non-psychopaths. For instance, Marcoux et al. (2014) found a higher pain threshold in people with psychopathic tendencies.

### Electrophysiology in Pain Research

Electrophysiological techniques, such as EEG, can discriminate event-related activity with a high temporal resolution and are therefore excellent methods to study if and when differences in neural signals related to certain events occur. Extracting such event-related activity from the ongoing electroencephalogram (EEG) allows researchers to study event-related potentials (ERPs). ERPs can be elicited by either actions, simple or complex stimuli, or events. This ERP technique allows us to directly study the neural responses associated with specific aspects of emotion and information processing.

#### Response-Locked ERPs

A specific component of the response-locked ERP that is studied in empathy-related research is the error-related negativity (ERN), which is an ERP that is associated with an incorrect motor response (e.g., a button press). It starts shortly before the time of an incorrect response and peaks around 100 ms thereafter (Falkenstein et al., 1991; Gehring et al., 1993). The ERN is generated within or near the dorsal ACC (Dehaene et al., 1994). Electrophysiological evidence demonstrated an association between the ERN, as electrophysiological correlate of action monitoring, and empathy-related affective responding (Larson et al., 2010; Thoma and Bellebaum, 2012). According to different theories, the ERN reflects the error-detection process itself (Falkenstein et al., 1991), or an emotional response to the error (Bush et al., 2000). Regarding the latter, research showed that an increased ERN has been associated with, for instance, concern over the outcome of an event (Gehring et al., 2000; Gehring and Willoughby, 2002). In line, a diminished ERN has been associated with a lack of concern over the outcome of an event (Santesso and Segalowitz, 2009).

#### Visual ERPs

Visual stimuli result in a series of peaks in the EEG and thereby determine the visual ERP. Perhaps the most studied component with respect to a wide range of cognitive processes is the P300 component (or P3). This visual P3 component is modulated by cognitive processes such as expectancy, relevance, meaning and attention (Gray et al., 2004). Several studies found that viewing painful stimuli caused a larger visual P3 amplitude over the posterior parietal area compared to viewing neutral pictures (Fan and Han, 2008; Meng J. et al., 2012).

#### Pain ERPs

Previous literature on empathy is mostly based on studies in which participants are not exposed to actual pain or pain in others directly (Singer et al., 2004; Botvinick et al., 2005). A more realistic, though controversial method, would be to introduce real-life situations of pain experience. Such experimental setups are not very common. One famous example stems from the controversial Milgram experiment that studied obedience (Milgram, 1963). In the current study, we adapted this approach to investigate the processing of painful stimuli delivered to oneself or to another person in both an active and a passive condition. Electrophysiological methods are useful in obtaining objective measures of clinically and experimentally induced pain and have proven to be successful in characterizing ERPs elicited by painful stimuli (Iannetti et al., 2005). Previous studies reporting pain ERPs describe an ERP that consists of a negative wave followed by a large positive wave that occurs *ca*. 400 ms after pain onset (Iannetti et al., 2005; Vossen H. G. M. et al., 2011). This positive peak has been labeled differently by several studies, for instance as a P2 (Iannetti et al., 2005) or as a P3 (Vossen H. et al., 2011). In addition, this late positive component has been reported to be increased when the subjective pain experience is more intense and is generated by the cingulate gyrus (Iannetti et al., 2005). Therefore, it has been proposed that this component can be used as an objective measure of experienced pain (Chen et al., 1979; Bromm, 1995).

### The Present Study

The present study investigated pain- and empathy-related neuronal responses in a socially interactive setup. The main aim of this experiment was to investigate the differences in neuronal responses with respect to the participants’ role, when observing someone in pain (villain) or receiving a painful stimulus (victim) and their capacity, when actively controlling the painful stimulus (active) or having no control over the painful stimulus (passive). Therefore, we designed a paradigm that included four conditions. During the first condition (“passive villain”) the participant passively watched another person pressing a button. During the second condition (“active villain”) the participant had to press the button him- or herself. During the third condition (“active victim”) the participant received the electrical shocks after pressing the button him- or herself and during the fourth condition (“passive victim”) another person was pressing the button while the participant was receiving the electrical shocks. In addition, we asked participants to fill in a self-report questionnaire to measure psychopathic traits in order to investigate the role of psychopathic personality traits on pain- and empathy-related neuronal responses.

We studied four contrasts in this paradigm. The first contrast compared the ERN of the response-locked ERP of the active villain vs. the active victim. This enabled us to study the amount of conflict the participant is experiencing when hurting himself or another person. Based on the fact that humans are social animals and are capable of showing empathy towards others, we expected the “active villain” to show an increased ERN compared to the “active victim”. Since psychopaths are characterized with low empathy, we expected that this effect correlated negatively with psychopathic traits.

The second contrast considered the potential difference between passive and active observing of another person in pain. The visual P3 component of the visual ERP of the passive villain vs. active villain were compared. We expected a higher visual P3 component for the active villain compared to the passive villain condition, since the active role creates a more involved and responsible position for the villain. In line, we expected a negative correlation of psychopathic traits with the magnitude of the visual P3 effect.

The third contrast compared the P3 component of the visual ERP of the active victim vs. the passive victim. This enabled us to study the role of having control over pain. Losing control over a threatening situation increases attention/vigilance which results in an increased visual P3 component. Therefore we expected to find higher visual P3 components for the passive victim compared to the active victim. We did not expect this contrast to be linked to psychopathic traits.

Also the fourth contrast is related to control over pain. We compared the late positive pain component of the pain ERP of the active victim vs. the passive victim. It has been demonstrated that pain is perceived as less intense when it is self-controlled (Pellino and Ward, 1998). This effect is reflected in attenuated neural responses in reaction to self-controlled pain (Salomons et al., 2004). Therefore, we expected to find an increased late positive pain component for the passive victim compared to the active victim. We did not expect this contrast to be linked to psychopathic traits.

Social neurocognition is a relatively new emerging field of social cognitive neuroscience. First, the current study provides insight on the influence of social context, attention and control over pain in self and others. Second, it enables us to better understand the role of psychopathic personality traits on social neurocognition. Third, the paradigm that was designed for this study provides as an alternative, more realistic method to study pain- and empathy-related behaviors.

## Materials and Methods

### Participants

A total of 60 healthy volunteers (31 females) with an age between 18 and 56 (*M* = 31.57, SD = 8.21) participated during a science fair: The Discovery Festival in Science Centre NEMO, Amsterdam, Netherlands in September 2015. Before actual participation in the experiment, participants were subjected to a test trial to introduce the nociceptive electrical stimulus. Participants that signed up for the study provided written informed consent and for each participant a short medical checklist was filled out by the researcher. Procedures were approved by the Ethics Committee Social Sciences (registered under amendment ECG2012-1301-010a2) of the Radboud University Nijmegen, Netherlands. Participants did not receive compensation for participation in this study and participants could leave the experiment at any time. One participant did not complete the whole experiment due to oversensitivity to the stimulation and two participants only completed two out of four conditions of the experiment. In addition, the EEG data of two participants contained excessive artifacts. Data of these five participants were excluded. The data of the remaining 55 participants (29 female; 9 left handed; age *M* = 31.8, SD = 8.00) were further analyzed.

### Self-Report Psychopathy Short-Form (SRP-SF)

Before the start of the ERP experiment, participants were asked to fill out the Self-Report Psychopathy Short Form (SRP-SF). The Self-Report Psychopathy (SRP) scale is designed to assess psychopathic traits in an adult non-forensic sample (Hare, 1985). The present study used a Dutch translation of the short version of the SRP (SRP-SF) that included 29 of the 64 original questions. The SRP-SF is highly correlated (*r* = 0.92) with the full version SRP (Paulhus et al., 2009) and has been proven to be valid and invariant across gender (Neumann and Pardini, 2014). The SRP-SF consists of two factors with each two subscales. Factor 1 (F1) covers interpersonal manipulation (e.g., “Sometimes you need to pretend that you like someone to get what you want”) and affective callousness (e.g., “Most people are weak”) and Factor 2 (F2) covers erratic lifestyle (e.g., “I”ve often done dangerous things just for the thrill’) and overt antisociality (e.g., “Sometimes I carry a weapon (knife or gun) to protect myself”). Questions needed to be rated on a 5-point Likert scale (1 = strongly disagree, 5 = strongly agree).

### ERP Paradigm

**Figure 1:**
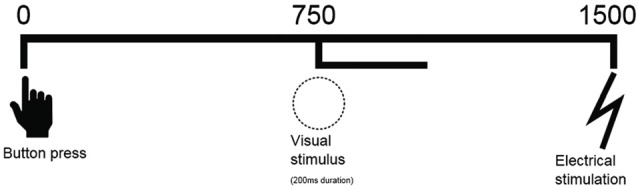
Schematic representation of sequence of events. A button press is followed by a visual stimulus on the screen (750 ms) for 200 ms and an electrical shock (1500 ms).

**Figure 2:**
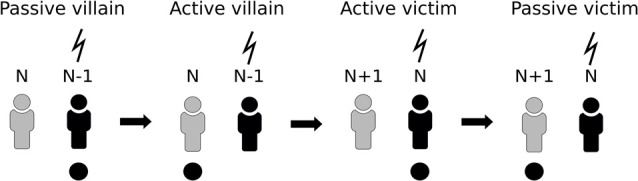
Schematic representation of the paradigm. Participant N undergoes all four conditions. In the first two conditions it acts as villain and then switches to victim, which is accompanied by N − 1 leaving the task and N + 1 entering.

Two participants were involved in the task at the same time and EEG of both participants was recorded during the whole experiment. The experiment included four conditions, each consisting of 15 trials. During the first condition (“passive villain”) participant N and participant N − 1 were seated next to each other while facing the same computer screen. Participant N − 1 was instructed to press a large red button which, after 750 ms, led to a 200 ms-presentation of a visual stimulus (white circle on a black background). The nociceptive electrical stimulus was delivered 750 ms after the onset of the visual stimulus to the left hand of participant N − 1 (Figure 1). During the second condition of the experiment (“active villain”) the roles for pushing the button were switched. Participant N was instructed to press the button while Participant N − 1 still received the electrical stimulus. In third condition (“active victim”) participant N was moved to the location of participant N − 1 who would now leave the experiment. A new participant, Participant N + 1, was introduced in the experiment starting with condition 1. Participant N was instructed to press the button which, after stimulus presentation, resulted in the electrical stimulus at his/her own arm while Participant N + 1 was observing. In the last condition (“passive victim”) participant N and participant N + 1 switched roles for pressing the button. Participant N + 1 was instructed to press the button while Participant N received the electrical stimulus. For a schematic representation of the design, see Figure 2.

Thus, each participant completed all four conditions. We chose not to randomize the different conditions, since, in line with the shared representation model (Decety and Jackson, 2004), previous pain experience or observation of pain in others could influence later pain experience or pain observation in others (Meng W. et al., 2012; Meng et al., 2013).

### EEG Recordings

All measurements were obtained using two mobile EEG labs. EEG and electro-oculography (EOG) signals were recorded with an actiCap-system which uses active Ag/AgCl electrodes (Brain Products GmbH, Munich, Germany). The Fz, Cz and Pz electrodes were placed according to the international 10-20 system, with an additional electrode on the right mastoid bone, the ground electrode at AFz, and the reference electrode over the left mastoid bone with self-adhesive rings. Post-recording, the electrodes were rereferenced to linked mastoids and filtered between 0.1 Hz and 30 Hz. Electrode impedance was kept below 20 kΩ which is appropriate for active electrodes (Mathewson et al., 2017). Eye movements were recorded by electrodes placed below the left eye and at the outer canthus of the left eye. The signal was digitized at 1000 Hz.

### Stimuli

The response-locked ERNs were captured when a large red button was pressed (diameter: 9.5 cm; height: 5.5 cm), visual ERPs were time-locked to the presentation of a visual warning stimulus (white circle on a black background) for 200 ms and pain ERPs were elicited by electric stimuli.

The electrical stimulation was delivered on the volar side of the non-dominant forearm by a concentric ring-electrode (Katsarava et al., 2006) attached to a Digitimer DS7-AH electrical stimulator (Digitimer Ltd). The participant received in total 30 electrical stimuli across both victim conditions, where each stimulus consisted of a rapid train of seven pulses with a 2 ms duration and a 2 ms inter-pulse-interval. Stimulus intensity was set to correspond to a perceived intensity of 7 on a scale of 0–10, where “0” corresponds with “I don’t feel anything”, and “10” corresponds with “maximum tolerated pain”) beforehand and was kept consistent throughout the experiment. Participants were exposed to short series of test stimuli after which they decided to participate in our experiment. All participants included in the analysis tolerated the painful stimulation.

### EEG Analyses

The segments belonging to the response, the visual stimulus and the nociceptive stimulus were selected offline. Epochs were defined as ranging from −250 ms to 750 ms based on stimulus or response markers for each of the three events. Baseline correction was applied using the interval of −250 ms to 0 ms. To allow blind scoring, component amplitudes were defined as the averaged value within a fixed latency window: The ERN component (20–70 ms), the visual P3 component (410–460 ms), the late positive pain P_400–500_ component (400–500 ms). After visual inspection of the grand average ERPs, the ERN, the visual P3 and pain P_400–500_ could be identified. Amplitudes of these components were determined as the average value within a fixed latency window (Picton et al., 2000). Segments were corrected for EOG artifacts by employing the Gratton and Coles algorithm (Woltering et al., 2013). In contrast to Woltering et al. (2013), averaging subtraction was not applied in the current analysis, which left the ERP components of interest unaffected. Trials contaminated with artifacts exceeding 150 μV were excluded. From the total amount of 825 trials that were measured during the experiment, 797 trials were included for further analysis. A 250 ms interval was used for baseline correction and response-locked, visual and pain ERPs were subsequently averaged per stimulus type. By averaging, all relevant ERP components were extracted from the ongoing signal according to Table 1.

**Table 1:** Schematic representation of the conditions, the event-related potentials and the contrasts.

	Passive villain	Active villain	Active victim	Passive victim
Motor response		ERN	ERN	
Interval (ms)		20–70	20–70	
Visual stimulus	visual P3	visual P3	visual P3	visual P3
Interval (ms)	410–460	410–460	410–460	410–460
Electrical shock			pain P_400–500_	pain P_400–500_
Interval (ms)			400–500	400–500

*Note. Horizontal: conditions, Vertical: event-related potentials*.

### Statistical Analyses

The ERN, the visual P3, and the pain P_400–500_ component amplitudes at Fz, Cz and Pz were further analyzed using repeated measures GLMs. The ERN and the pain P_400–500_ were analyzed using a 2-by-3 design; capacity (passive/active) or role (villain/victim) and electrode site (Fz, Cz and Pz) functioned as within-subject variables. The visual P3 was analyzed using a 2-by-2-by-3 design, as all four conditions were included, which cover two potential roles (villain/victim) in an active as well as a passive capacity (also see Table 1). Greenhouse-Geisser correction was applied when the sphericity assumption was violated. The significance level was set at *α* < 0.05. Since the hypotheses concerning the contrasts were formulated* a priori*, no correction of the *p*-values was required.

Furthermore, we studied the correlations of the total scores and the subscales of the SRP with the difference scores of the contrasts. Difference scores of the contrasts were calculated by subtracting the control condition (passive villain/active victim) from the experimental condition (active villain/passive victim). All statistical analyses were performed in IBM SPSS Statistics Version 22.

## Results

**Figure 3:**
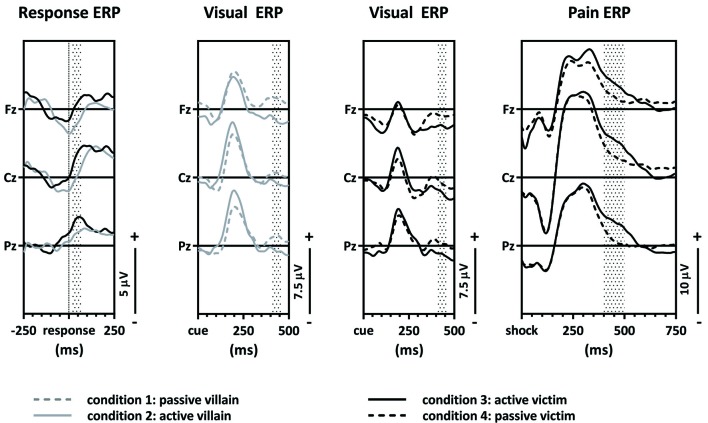
Grand average event-related potentials (ERPs). The response-locked ERPs of the active villain vs. active victim, the visual ERPs of the passive villain vs. active villain, the visual ERPs of the active victim vs. passive victim and pain ERPs of the active victim vs. passive victim.

Grand average response-locked, visual and pain ERPs were constructed (Figure 3). An average of 4.1% (SD = 2.8%) of trials was excluded due to contamination with artifacts (breakdowns per trial type are in Supplementary Materials Table S4). A complete overview of the main effects of the contrasts and the correlations with SRP questionnaire is shown in the Supplementary Materials Tables S2, S3.

### The ERN Component of the Response-Locked ERPs

**Figure 4:**
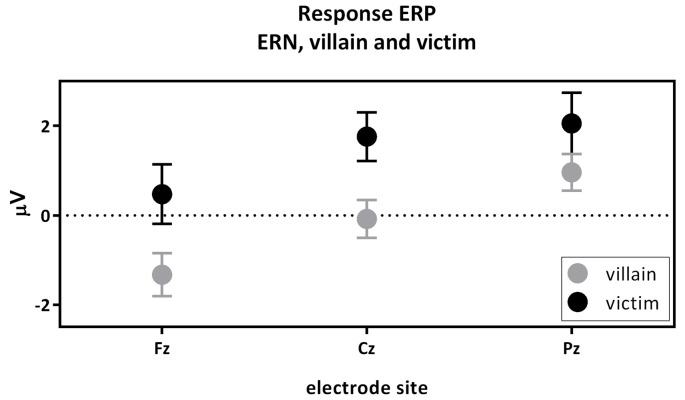
The response-locked error-related negativity (ERN) active villain vs. active victim. ERN amplitude (μV) of the midline electrodes (Fz, Cz, Pz) are displayed. The ERN was significantly more negative for the villain than the victim (partial eta^2^ = 0.102; *p* = 0.016).

The first contrast compared the ERN of the active villain and the active victim (Figure 4). A significant main effect for role was found where the ERN was more negative for the villain than the victim (*F*_(1,54)_ = 6.15; *p* = 0.016; partial eta^2^ = 0.102; Figure 3: Response ERP). As expected, a main effect for electrode was found (*F*_(1.53,82.83)_ = 13.19; *p* < 0.001; partial eta^2^ = 0.196). No interaction effect was observed between role and electrode (*F*_(1.61,86,83)_ = 1.534; *p* = 0.223; partial eta^2^ = 0.028). The ERN was maximal at the Fz electrode, therefore the magnitude of the ERN difference at Fz was used to calculate the correlations with psychopathic traits. However, there were no significant correlations with psychopathic traits.

### The P3 Component of the Visual ERPs

Both the second and third contrasts were tested using a single (2-by-2-by-3) overall GLM, which showed an effect of role (villain/victim; *F*_(1,54)_ = 5.446; *p* = 0.023; partial eta^2^ = 0.092) as well as an effect of capacity (active/passive; *F*_(1,54)_ = 9.223; *p* = 0.004; partial eta^2^ = 0.146) on the P3. No interaction between role and capacity was present(*F*_(1,54)_ = 0.794; *p* = 0.377; partial eta^2^ = 0.014), nor was a three-way interaction apparent (*F*_(2,108)_ = 1.031; *p* = 0.360; partial eta^2^ = 0.019). This means that the effect of capacity was present in both roles, which then relates directly to the two contrasts (contrast two and contrast three). A significant effect of electrode was found (*F*_(1.76,95,22)_ = 3.611; *p* = 0.030; partial eta^2^ = 0.063), with the P3 being maximal at Pz. Therefore, Pz was used for further analysis. There was no interaction effect of electrode with capacity (*F*_(1.77,95.69)_ = 1.345; *p* = 0.265; partial eta^2^ = 0.024) or role (*F*_(2,108)_ = 0.804; *p* = 0.450; partial eta^2^ = 0.015).

**Figure 5:**
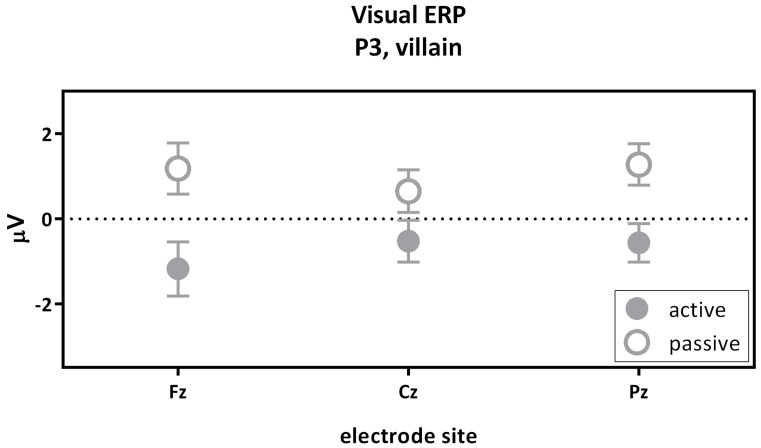
The visual P3 passive villain vs. active villain. Visual P3 amplitude (μV) of the midline electrodes (Fz, Cz, Pz) are displayed. The visual P3 was decreased for the active villain compared to the passive villain (partial eta^2^ = 0.116; *p* = 0.010).

The second contrast compared the visual P3 of the passive and the active villain (Figure 5). A main effect of capacity was found for the P3, as showed by the overall analysis given above. The visual P3 was decreased for the active villain compared to the passive villain (Figure 3: Visual ERP). There were no significant correlations with psychopathic traits.

**Figure 6:**
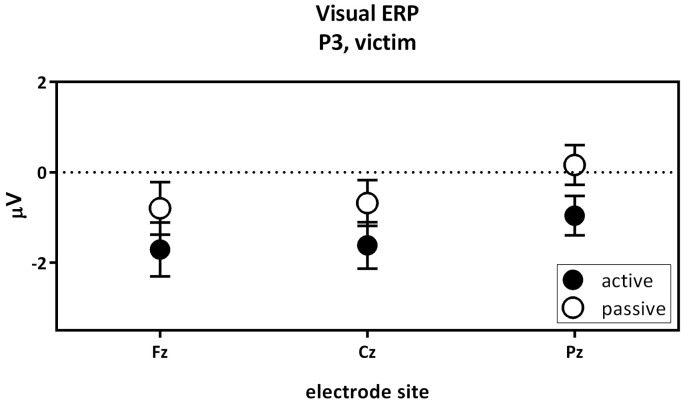
The visual P3 passive victim vs. active victim. Visual P3 amplitude (μV) of the midline electrodes (Fz, Cz, Pz) are displayed. The visual P3 was decreased for the active victim compared to the passive victim.

The third contrast compared the visual P3 of the active victim and the passive victim (Figure 6). According to the overall GLM, a main effect for capacity was found. The visual P3 was decreased for the active victim compared to the passive victim (Figure 3: Visual ERP). There were no significant correlations with psychopathic traits.

### The P_**400–500**_ Component of the Pain ERPs

**Figure 7:**
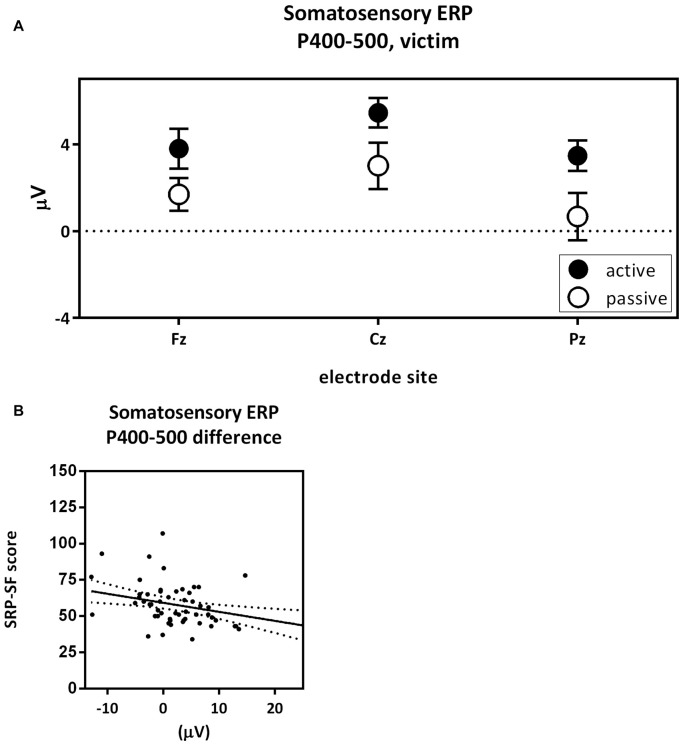
Pain P_400–500_ passive victim vs. active victim. P_400–500_ amplitude (μV) of the midline electrodes (Fz, Cz, Pz) are displayed in **(A)**. The pain P_400–500_ was decreased for the passive victim compared to the active victim (partial eta^2^ = 0.082; *p* = 0.033). Scatterplots of the correlations with the total Self-Report Psychopathy (SRP) score (*r* = −0.370; *p* < 0.005) are shown in **(B)**.

The fourth contrast compared the pain P_400–500_ of the active victim and passive victim (Figure 7A). There was a significant main effect for capacity (*F*_(1,54)_ = 4.81; *p* = 0.033; partial eta^2^ = 0.082) where the pain P_400–500_ was decreased for the passive victim compared to the active victim (Figure 3: Pain ERP). Moreover, there was a main effect for electrode (*F*_(1.36,73.41)_ = 7.59; *p* = 0.004; partial eta^2^ = 0.123). The pain P_400–500_ was maximal at the Pz electrode, therefore Pz was used for further analysis. There was no interaction effect for electrode and capacity (*F*_(1.42,76.41)_ = 0.473; *p* = 0.625; partial eta^2^ = 0.009).

The difference score of the pain P_400–500_ was negatively correlated with the total score on the SRP (*r* = −0.370; *p* = 0.005; Figure 7B). More specifically F1 scores of the SRP negatively correlated with difference scores of the pain P_400–500_ (*r* = −0.328; *p* = 0.015) and the interpersonal subscale seemed to play an important role (*r* = −0.321; *p* = 0.017). F2 scores of the SRP negatively correlated with difference scores of the pain P_400–500_ (*r* = −0.343; *p* = 0.010), where the lifestyle subscale seemed to play an important role (*r* = −0.412; *p* = 0.002).

## Discussion

The current study investigated the neural responses of pain- and empathy-related processes in a social, EEG-coupled paradigm. Moreover, we were interested in possible links with psychopathic traits. The first contrast compared the ERNs resulting from the button press of the active villain and the active victim. A clear ERN appeared in response to inflicting pain. Since the ERN is related to conflict, this finding suggests that people experience conflict when hurting themselves or someone else. This outcome is consistent with other findings on empathy (Avenanti et al., 2005; Bufalari et al., 2007; Singer and Lamm, 2009; Decety, 2010). More specifically, our findings suggest that people experience more conflict when hurting someone else than when hurting themselves. To the best of our knowledge, this direct comparison between self-compassion and empathy for others was not made before. Although we would have expected that psychopathic traits correlate negatively with the ERN, we could not confirm this in the current study.

The second contrast compared the visual P3 components of the villain between the passive and active condition. Results indicated a higher visual P3 amplitude for the passive villain compared to the active villain. Previous findings suggested the amplitude of visual P3 to be larger for relevant stimuli than irrelevant stimuli (Steffensen et al., 2008). In the present study the visual stimulus predicting an upcoming shock seems more relevant for the passive villain than for the active villain. For the active villain, the button press already provides information about the upcoming electrical stimulus and the visual stimulus does not add any new information. For the passive villain, the stimulus provides new information. Therefore, we could conclude that our finding is in line with previous literature. This effect did not seem to be influenced by psychopathic traits as no significant correlations were observed.

The third contrast compared the visual P3 components of the active and the passive victim. As expected, results indicated an increase in visual P3 amplitude for the passive victim compared to the active victim. The loss over control over the shock leads to heightened attention or vigilance in the passive condition. This effect did not seem to be influenced by psychopathic traits as no significant correlations were observed.

The fourth contrast compared the pain P_400–500_ components of the active and the passive victim. Results showed an increased pain P_400–500_ amplitude for the active victim compared to the passive victim in response to the shock. This finding is contradicting other studies that suggest that self-controlled pain is perceived as less intense (Pellino and Ward, 1998). A more recent study showed that less predictable pain has a larger impact. However, this is not expressed in pain experience but in physiological impact (heart rate, reaction times) and primary tasks (Arntz and Hopmans, 1998). Moreover, pain literature suggests that attention to pain increases the perceived pain intensity (Villemure and Bushnell, 2009). Actively attributing pain to oneself could heighten attention during the trial, thus also for receiving the shock, and therefore explain the increased pain P_400–500_ in the active victim in the current study. Besides, the SRP negatively correlated with the pain P_400–500_ difference score. This suggests that the more psychopathic traits, the less different the pain is experienced in a situation in which the shock is delivered by themselves compared to a situation in which the shock is delivered by another person. Possibly, painful stimuli might be perceived as being less salient for people scoring high compared to people scoring low on psychopathic traits, and painful stimuli might therefore attract less attention with increased psychopathic traits in both conditions.

In all, recent findings indicate that people experience more conflict when hurting others than when hurting themselves. Furthermore, the results indicate that self-controlled pain was experienced as more painful than uncontrolled pain, which contradicts earlier findings in pain research. Besides, findings showed that people that scored high on psychopathic traits seemed to attend to and experience pain differently. Based on these findings, we suggest that social context, attention and personality traits are important modulators of pain- and empathy-related neuronal responses. Pain experience can be modulated by attention and the way that pain is controlled (self or other). Relevance of being in control or not, the processing of pain predicting stimuli, the salience of such stimuli and attention directed towards these stimuli are all important modulators of empathy-related neuronal responses. In line, psychopathic traits, and indirectly empathic traits, affect pain-related neuronal responses.

When interpreting the results, we should take into account that the sequence of conditions was equal for all participants based on ethical considerations. We encountered the order effect of first undergoing the villain conditions followed by the victim conditions. Two distinct forms of perspective taking are described as the “imagine-other” and “imagine-self” perspective. Where “imagine-other” perspective describes the situation in which someone imagines how the other perceives a certain situation and how the other feels as a result, the “image-self” perspective describes the situation in which you imagine how you would perceive a certain situation, were you in the other’s position and how you would feel as a result (Batson et al., 1997). The present study measures the imagine-self perspective during the villain conditions, since the villain is aware of the fact that he will be put in the position of the victim afterwards. This could be beneficial because participants experience feelings of distress during the villain conditions (Batson et al., 1997) and this may lead to stronger effects during all conditions. However, the order effect might also be seen as a limitation. Since the active victim condition is always first, this could result in a habituation effect for the passive victim.

Another limitation is that the inclusion criteria were lenient. For instance, age was not restricted and from previous literature we learned that older participants show longer P3 latencies (Mullis et al., 1985; Kuba et al., 2012). Moreover, the experiment was done overnight at a festival. These limitations were mostly controlled by the within-subject design and even though this experiment was performed in a semi-controlled environment, we found robust effects that were overall in line with previous literature.

All in all, we suggest that both pain- and empathy-related neuronal responses are modulated by social context, attention and personality traits. Moreover, the within-subject experimental design described in this study thus provides an excellent approach to further unravel the influence of personality traits on social cognition.

## Author Contributions

CHH: writing, final data analysis. JMAD: writing, final data analysis, study set-up. MLAJ: study set up, final data analysis, writing. CMR: study set up, data-analysis, writing. MA, MNB, PB, LB-B, JF-C, MH, ACJ, LL-B, CS, LRV-A, RHAW: study set up, data acquisition, data-analysis.

## Conflict of Interest Statement

The authors declare that the research was conducted in the absence of any commercial or financial relationships that could be construed as a potential conflict of interest.
