# Supplementary material for: Pain Processing in a Social Context and the Link with Psychopathic Personality Traits—An Event-Related Potential Study
Source: Front Behav Neurosci. 2017 Sep 25;11:180. doi: 10.3389/fnbeh.2017.00180 (PMC5622147; doi:10.3389/fnbeh.2017.00180)
Supplement: Supplementary file 1 [file Data_Sheet_1.docx]

Supplementary Material

**Pain Processing in a Social Context and the Link with Psychopathic Personality Traits**

***Casper H. van Heck^#1^, Josi M. A. Driessen^#^*^1,2^, Maria Amato^1^, Marnou N. van den Berg^1^, Pritha Bhandari^1^, Laura Bilbao-Broch^1^, Jordi Farres-Casals^1^, Manon Hendriks^1^, Adrian C. Jodzio^1^, Laura Luque-Ballesteros^1^, Christina Schöchl^1^, Laura R. Velasco-Angeles^1^, Roel H. A. Weijer^1^, Clementina M . van Rijn^1^ & Marijtje L. A. Jongsma^3^***

^#^These authors contributed equally to this study

*** Correspondence:** Josi Driessen: j.driessen@donders.ru.nl

# Supplementary Data

**Supplementary material**

| **Table 2. Main effects of the contrasts** | | | |
| --- | --- | --- | --- |
|  | F | P-value | *partial η^2^* |
| Fz **response ERP ERN** | 6,151 | 0,016* | 0,102 |
| active victim vs. active villain |  |  |  |
| Pz **visual ERP P3** | 9,233 | 0,004* | 0,146 |
| passive villain vs. active villain |  |  |  |
| Pz **visual ERP P3** | 9,233 | 0,004* | 0,146 |
| active victim vs. passive victim |  |  |  |
| Pz **pain ERP P3** | 4,806 | 0,033* | 0,082 |
| active victim vs. passive victim |  |  |  |
| *Note. Significant values are marked with an asterisk* | | | |

| **Table 3. Correlations of contrast with SRP outcomes** | | | | | | | | |
| --- | --- | --- | --- | --- | --- | --- | --- | --- |
|  | | Interpersonal | Affective | Lifestyle | Antisocial | F1 traits | F2 traits | Total |
| Fz **response ERP** **ERN** | Pearson Corr. | -0,134 | 0,041 | 0,083 | 0,009 | 0,003 | 0,057 | 0,031 |
| active victim vs. active villain | Sig. (2-tailed) | 0,807 | 0,764 | 0,548 | 0,949 | 0,985 | 0,6811 | 0,821 |
| Pz **visual ERP P3** | Pearson Corr. | -0,060 | -0,052 | 0,075 | 0,065 | -0,062 | 0,077 | 0,004 |
| passive villain vs. active villain | Sig. (2-tailed) | 0,665 | 0,706 | 0,587 | 0,636 | 0,651 | 0,577 | 0,977 |
| Pz **visual ERP P3** | Pearson Corr. | 0,065 | 0,120 | 0,082 | -0,047 | 0,102 | 0,031 | 0,075 |
| passive victim vs. active victim | Sig. (2-tailed) | 0,637 | 0,382 | 0,554 | 0,733 | 0,459 | 0,823 | 0,585 |
| Pz **pain ERP P3** | Pearson Corr. | -0,321* | -0,264 | -0,412** | -0,179 | -0,328* | -0,343* | -0,370** |
| active victim vs. passive victim | Sig. (2-tailed) | 0,017 | 0,051 | 0,002 | 0,190 | 0,015 | 0,010 | 0,005 |

| **Table 4. Rejected trials (%) per condition** | | | |
| --- | --- | --- | --- |
| **Component** | **Role** | **Task** | **Rejected trials** |
| Response ERP | Villain | Active | 1.3% |
| Response ERP | Victim | Active | 3.0% |
| Visual ERP | Villain | Passive | 3.0% |
| Visual ERP | Villain | Active | 2.0% |
| Visual ERP | Victim | Active | 3.8% |
| Visual ERP | Victim | Passive | 3.8% |
| Pain ERP | Victim | Active | 10.4% |
| Pain ERP | Victim | Passive | 5.5% |
